# Contraception in adolescents with mental disorders: adherence and satisfaction in the use of depot medroxyprogesterone acetate

**DOI:** 10.61622/rbgo/2025rbgo9

**Published:** 2025-03-17

**Authors:** Giani Silvana Schwengber Cezimbra, Edward Araujo Júnior, Cristina Aparecida Falbo Guazzelli

**Affiliations:** 1 Universidade Federal de São Paulo Escola Paulista de Medicina Department of Obstetrics São Paulo SP Brazil Department of Obstetrics, Escola Paulista de Medicina, Universidade Federal de São Paulo, São Paulo, SP, Brazil.

**Keywords:** Pregnancy in adolescence, Pregnancy, Mental disorders, Contraception, Medroxyprogesterone acetate

## Abstract

**Objective:**

To evaluate the continuation rate, satisfaction, and reasons for discontinuation of depot medroxyprogesterone acetate (DMPA) in adolescents treated in a mental health service.

**Methods:**

Prospective cohort study conducted in a reference unit for the care of adolescents with mental disorders (MDs) and intellectual disabilities (IDs). All patients received a gynecological consultation and an educational group on contraceptive methods. Sociodemographic data on age, education and gynecological data (menarche, coitarche, regularity of menstrual cycles and presence of symptoms) were collected. Follow-up was quarterly for 12 months, during which symptoms, desire to continue, and satisfaction with the use of the quarterly injectable were assessed.

**Results:**

Eight hundred and sixty-two sexually active adolescents were supported, 532 adolescents chose to use the quarterly injectable, and 69 of these agreed to participate in the study. The mean age of users was 15.5 years (SD=0.91). After 12 months of follow-up, 34 (49.3%) of the 69 adolescents continued to use the method and 36 (52.3%) were satisfied. Among the 33 (47.8%) who discontinued use, the most common reasons were irregular bleeding and weight gain.

**Conclusions:**

Adolescents with intellectual disabilities and/or other mental disorders showed a significant rate of continuation and satisfaction with the use of the depot medroxyprogesterone acetate at 12 months, and the most common reasons for discontinuation were irregular uterine bleeding and weight gain.

## Introduction

Adolescents with mental disorders (MDs) and intellectual disabilities (IDs) almost always become pregnant unplanned and are more vulnerable to this risk than adolescents without the condition.^(1)^ Some factors that contribute to this occurrence are lack of knowledge in contraception methods, impulsivity and lack of behavioral control. Difficulties in regular contraceptive use and possible drug interactions are also worth mentioning.^(2-4)^

Reproductive health problems, such as pubertal characteristics, sexuality and menstrual difficulties, may be more common in these adolescents and their families. This period is associated with concerns about menstrual hygiene, risk of abuse, vulnerability and mood changes.^(5)^

Adolescents with MDs often use psychotropic drugs, which can be teratogenic and increase the risk of preterm birth.^(6)^ The most recommended methods for these adolescents are LARCS, especially hormonal intrauterine devices and subcutaneous implants, as well as depot medroxyprogesterone acetate (DMPA), considering the benefit of reducing bleeding in this population.^(5)^ The American College of Obstetricians and Gynecologists (ACOG) recommends the use of DMPA to promote a reduction in blood flow, amenorrhea and perimenstrual symptoms.^(7)^ The use of DMPA has been widely recommended due to its high efficacy, ease of dosing and safety.^(8)^ The choice of DMPA in this study is due to its frequent use in recent decades for contraception in adolescents with mental disorders.^(9)^ There are few studies focusing on reproductive planning and contraception in this population with its specificities.

The aim of this study was to evaluate the continuation rate, satisfaction and reasons for discontinuation of DMPA in adolescents treated in a mental health service.

## Methods

This study was a prospective cohort conducted in a reference unit for the care of adolescents (aged 10-19 years) with mild to moderate intensity MDs and their families, the ADOLESCENTRO (*Secretaria Estadual de Saúde / Distrito Federal*), Brasília, DF, Brazil. All patients who received a gynecological consultation and an educational group on contraceptive methods were invited to participate in the study.

The inclusion criteria were female adolescents in need and desire of contraception, between 10 and 19 years of age, with sexual activity for contraception, and with IDs and other mild to moderate MDs, according to the criteria of the Diagnostic and Statistical Manual of Mental Disorders 5th edition (DSM-5),^(10)^ in treatment. Adolescents with mild cases of IDs were included in the study based on their ability to understand, make choices, and function in general.

Among the exclusion criteria were: presenting any contraindication listed in the package insert of the chosen method; presenting any systemic disease such as liver disease, coagulopathy, cancer or any other factor that contraindicates the use of the chosen method; presenting any gynecological disease such as uterine malformation, vaginal bleeding without etiological diagnosis or any other factor that contraindicates the use of the chosen method; not demonstrating cognitive conditions to understand their contraceptive choice.

At baseline, sociodemographic data on age, education, and gynecological data were collected. Information on medical history and medications used was recorded. The adolescents were followed for 12 months and evaluated every 3 months. At the return visits, clinical assessments were performed, including weight and blood pressure measurements.

This study was approved by the Research Ethics Committee of the Federal University of São Paulo (UNIFESP), number: 2.659.135, on May 16, 2018, and the participants signed the informed consent form.

## Results

**Figure 1:**
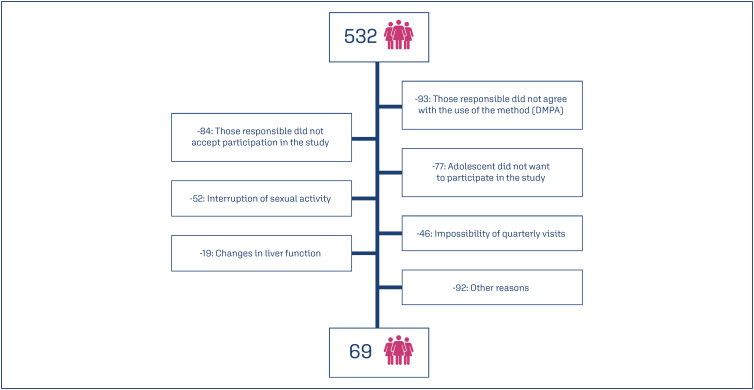
Flowchart of the included patients

After attending an educational group, the choice of method was made freely by the adolescent with the help of her guardian and evaluated with the gynecologist according to her needs and indications. A total of 532 adolescents who were interested in using the quarterly injectable method were recruited, of whom 69 agreed to participate in the study. Figure 1 shows the flowchart of the included patients.

Sociodemographic and other characteristics are shown in table 1. The mean age was 15.4 years (SD: 0.91). Among the data collected, we observed a high rate (60.8%) of academic delay, represented by the low number of years of study. This data was evaluated according to the study subjects in relation to the age they were at the time of the research and the number of years of education they should have had if they had not failed or dropped out. Another finding that draws attention is the incidence of sexual violence in this group (49%). Notification is always made in the Violence and Accident Surveillance System (Viva/Sinan) and they are taken for evaluation and psychotherapeutic, medication and other treatment, when necessary. The most common MDs were: depression (30.10%), anxiety (22.20%), epilepsy (9.50%), bipolar affective disorder (7.90%), attention deficit hyperactivity disorder (7.90%), attempted suicide (7.90%) and eating disorders (6.3%). IDs were present in 9.5% of cases. Of the 69 participants, 57 (82.6%) were taking psychiatric medication at baseline and 12 (17.4%) were receiving psychotherapy (Table 1).

**Table 1:** Demographic characteristics of the 69 adolescents

Data		Mean	SD
Age (years)		15.4	0.91
Sexual initiation (years)		14.2	1.52
Menarche (years)		11.7	1.50
**Education (years of study)**		**n(%)**	
	≤ 9	42(60.8)	
	9 to 12	27(39.2)	
Previous pregnancy			
	None	68(98.5)	
	One	1(1.5)	
Sexual violence			
	Yes	34(49)	
	No	35(51)	
Mental disorder treatment			
	Medication	57(82.6)	
	Psychotherapy	12(17.4)	

SD - standard deviation

The most common signs and symptoms associated with DMPA use were menstrual irregularity (43.4%) and weight gain (46.4%). Headache, edema, mood changes and pelvic pain were also reported. Regarding the bleeding pattern, 54% of them experienced amenorrhea after 12 months of use, and the most common complaints were characterized as prolonged or frequent bleeding (31.8%) and infrequent bleeding (11.6%), according to table 2.^(11)^

**Table 2:** Bleeding pattern and most frequent complaints in adolescent depot medroxyprogesterone acetate users

		n(%)
Blending pattern		
	Amenorrhea	37(54)
	Infrequent bleeding *	8(11.6)
	Prolonged or frequent bleeding **	22(31.8)
	Standard bleed ***	2(2.9)
Most frequent complaints		
	Weight gain ****	35(50.7)
	Prolonged or frequent bleeding	22(31.8)
	Mood changes ****	11(15.9)
	Edema	8(11.6)
	Headache *****	5(7.2)

* Infrequent bleeding: 2 or fewer times in 90 days.

** Prolonged or frequent bleeding: more than 5 times or lasting 14 days or more in 90 days.

*** Standard bleeding: 3 to 5 times in 90 days^11^

**** Data such as weight gain, mood changes and headache mentioned in this table may present biases due to the mental disorder itself and the psychiatric medications used.

Among the 32 (46.4%) adolescents who showed weight gain, the range was from 2 to 20 kg, with a mean of 6.5 kg (standard deviation - SD= 4.6 kg and 95% confidence interval - CI = 6.5 ± 1.59). Regarding continuation of the method, after 12 months of follow-up, 49.2% (34) continued to use DMPA, while 47.8% (33) stopped using it and 2.9% (2) discontinued. Satisfaction with the method was 52.3% among users (Table 3).

**Table 3:** Rate of continuity and satisfaction in adolescent depot medroxyprogesterone acetate (DMPA) users

DMPA		n(%)
Continuity		
	6 months	57(82.6)
	9 months	45(65.2)
	> 12 months	34(49.3)
	Dropout	2(2.9)
Satisfaction		
	Satisfied	36(52.3)
	Dissatisfied	31(44.9)
	Dropout	2(2.9)

## Discussion

The mean age of our population was 15.4 years (SD: 0.91) and sexual initiation was 14.2 years (SD: 1.52). These data show a delay in the introduction of contraception, considering the risk of pregnancy and that none of them had used any method before entering the study. These adolescents are patients who undergo many medical and psychological consultations, where there is no guidance on contraception. Other studies have linked this fact to the invisibility and inattention of parents and health professionals to the sexual and reproductive needs of these adolescents.^(12)^

DMPA is a well-accepted contraceptive method among adolescents with MDs or IDs due to its ease of dosing and the possible presence of amenorrhea. In addition, there is a non-contraceptive benefit due to its anticonvulsant effects, with the possibility of reducing the frequency and intensity of epileptic seizures.^(5)^ In our study, the most common adverse effects of DMPA use were weight gain (46.4%) and menstrual irregularities (43.4%). Some adolescents reported the presence of headache, edema and mood changes. These symptoms may often be associated with MDs or adverse effects of the medications used and were therefore not evaluated in this specific group.

Weight gain was observed in a large proportion (46.4%) of adolescents using the quarterly injectable, which is in line with several articles on weight gain in users of quarterly injectable DMPA, although there may be many other factors associated with this and many women are able to maintain their weight with dietary measures and physical activity.^(13,14)^ A recent study reported that DMPA users had significantly greater increases in weight (5.1 kg) and body fat (3.4%) over a period of time 3 years compared to women using non-hormonal or oral contraceptives.^(13)^

Appropriate counseling prior to initiation may improve the perception of weight change and reduce the discontinuation rate. On the other hand, it is important to note that factors related to the MD itself and the psychiatric medications used (such as lithium, quetiapine, and risperidone) may be associated with changes in appetite and weight in the adolescents studied.^(13)^ In the case of adolescents with MDs using psychiatric medications that may interfere with weight gain, we consider this an additional reason for the results obtained in this study.

Regarding the change in bleeding pattern, amenorrhea was observed in 37 (54%) of the adolescents. In most cases, amenorrhea was desired and well accepted by the adolescents and their caregivers, which influenced the level of acceptance and continuation. Among other bleeding changes, the main factors that suggests having motivated the early interruption of the method in this study was weight gain in 50.7% of the adolescents and the occurrence of prolonged or frequent bleeding in 31.8% of cases. These data were similar to other studies reporting the occurrence of irregular bleeding in 49% to 90% of patients.^(7)^

A relevant fact observed was the incidence of sexual violence in 49% of the population studied. In Brazil, according to *Instituto Brasileiro de Geografia e Estatística* (IBGE) data,^(15)^ between 2009 and 2019, one in five adolescents in general (20.1%) between the ages of 13 and 17 reported having experienced some form of sexual violence. An American study of high school students with physical disabilities or health problems found a higher incidence of forced sexual intercourse (19.6%) compared to adolescents without health problems (9.4%).^(16)^ These data indicate the increased vulnerability of this group to this type of violence.

In terms of continuity, of the 69 adolescents who started the study, 49.2% continued to use depot medroxyprogesterone acetate for 1 year or more. The most common reasons for early discontinuation among our adolescents were irregular bleeding (43.4%) and weight gain (46.4%). It is important to note that adolescents with MDs have other difficulties related to their specific mental condition that increase the risk of failure or discontinuation.^(3,14)^ In the literature, we found few studies evaluating the use of this method in adolescents with MDs. In disease-free adolescents participating in the CHOICE project, it was observed that among girls aged 14-19 years who used DMPA for 12 months, the continuation rate was 47.3%.^(17)^ In another 12-month longitudinal cohort study of 1,387 adolescents and young women aged 15-24 years attending public family planning clinics, the 12-month continuation rate with DMPA was 12.1%.^(18)^ Similar to our results, another study found that 45% of adolescents discontinued the method in the first year of use, mainly due to irregular bleeding and weight gain, data consistent with our study.^(19)^

In terms of satisfaction, after 12 months of use, 52.3% of adolescents were satisfied with DMPA use, although 2 of them discontinued use for other reasons. The most common reasons for continued use were safety, ease of dosing, and amenorrhea. In a study of 262 adolescents and young adults with special needs who wanted or needed to suppress menstruation, such as women with cerebral palsy and autism, or with gynecological conditions, it was shown that patients tended to have a high percentage of satisfaction (93.3%) with the use of DMPA due to its menstrual suppression effect.^(20)^

## Conclusion

Adolescents with MDS showed a significant rate of continuation and satisfaction with use of the quarterly injectable. Weight gain and irregular bleeding were the most common reasons for discontinuation. DMPA may be a contraceptive option for adolescents with MDs.
